# RNA Silencing in the Management of Dyslipidemias

**DOI:** 10.1007/s11883-021-00968-7

**Published:** 2021-09-01

**Authors:** Neil C. Henney, Maciej Banach, Peter E. Penson

**Affiliations:** 1 School of Pharmacy and Biomolecular Sciences, Liverpool John Moores University, Byrom Street, Liverpool, L3 3AF UK; 2 Liverpool Centre for Cardiovascular Science, Liverpool, UK; 3 Department of Hypertension, Chair of Nephrology and Hypertension, Medical University of Lodz, Lodz, Poland; 4 Cardiovascular Research Centre, University of Zielona Gora, Zielona Gora, Poland

**Keywords:** Inclisiran, LDL cholesterol, Dyslipidaemia, Atherosclerosis, siRNA

## Abstract

**Purpose of Review:**

Remarkable reductions in cardiovascular morbidity and mortality have been achieved in recent decades through the widespread use of ‘small-molecule’ hypolipidaemic drugs such as statins and ezetimibe. An alternative approach is to perturb the production of proteins through ribonucleic acid (RNA) silencing, leading to long-lasting knock-down of specific biological molecules. This review describes the scientific basis of RNA silencing, and critically evaluates the evidence relating to inclisiran, a small interfering RNA against proprotein convertase subtilisin kexin 9 (PCSK9).

**Recent Findings:**

Pooled analysis of three recent ORION trials has demonstrated that twice-yearly administration of inclisiran reduces LDL-C by 50% in a range of patient groups, with only mild adverse effects.

**Summary:**

Inclisiran provides safe, effective and long-lasting reductions in PCSK9 and LDL-C. The results of the phase-3 ORION-4 outcomes study are eagerly awaited. Further promising RNA silencing technologies have the potential to improve the management of dyslipidaemia.

## Introduction

Substantial reductions in cardiovascular morbidity and mortality have been achieved in recent decades through the widespread use of hypolipidaemic drugs, which act as enzyme inhibitors (statins) or modulate transporters (ezetimibe) or receptor proteins (fibrates) [1]. Whilst undoubtedly effective, these small-molecule agents have the disadvantages of off-target effects and require good compliance with daily dosing. An alternative approach is to perturb the production of proteins through ribonucleic acid (RNA) silencing, leading to long-lasting knock-down of specific biological molecules, thus overcoming the need for daily dosing and potentially avoiding off-target biological effects. This review will describe the scientific basis of RNA silencing and critically evaluate the evidence relating to inclisiran (a small interfering RNA against proprotein convertase subtilisin kexin 9 (PCSK9)).

## The clinical Need for New Pharmacological Approaches in Dyslipidaemia

Cardiovascular disease (CVD) prevention, and hypolipidaemic drugs, in particular, is a great successes story of modern medicine. Statins (which inhibit 3-hydroxy-3-methyl-glutaryl-coenzyme A (HMG-CoA) reductase, the rate-limiting step in the mevalonate pathway of hepatic cholesterol synthesis) are safe, well-tolerated, and reduce the risk of major cardiovascular events by about one quarter, for each mmol/l reduction in low-density lipoprotein cholesterol (LDL-C) [2]. Additional LDL-C lowering with ezetimibe (which blocks the intestinal NPC1-like intracellular cholesterol transporter 1, thereby reducing the absorption of dietary cholesterol) leads to a further reduction in cardiovascular risk [3]. However, a substantial proportion of treated individuals do not reach their LDL-C treatment targets [4], and these targets may be too liberal. An overwhelming body of evidence from intervention trials, observational studies and Mendelian randomisation shows that the risk of atherosclerotic cardiovascular events, including myocardial infarction and ischaemic stroke, is strongly associated with lifetime exposure to LDL-C [5, 6••], thus highlighting the importance of the concept that ‘Lower better longer’ with respect to LDL-C, and other lipoproteins containing apolipoprotein B [7]. Where optimal levels of LDL-C cannot be met with current therapies, new approaches are needed, both in primary prevention [4] and especially in the secondary prevention of cardiovascular disease, where the likelihood of recurrent disease is very high [8]. Exciting advances have occurred in this field, including the recent development of bempedoic acid, an orally active prodrug, which is activated specifically in the liver to inhibit ATP citrate lyase (an enzyme upstream of HMG-CoA reductase in the mevalonate pathway of cholesterol synthesis) [4]. Nevertheless, the ‘small-molecule’ approach to drug development is largely limited to biological targets with an enzymatic active site or known regulatory domain, whereas antibody-based approaches and RNA silencing are not [9].

Small molecules often have ‘off-target’ interactions resulting in adverse effects. In the case of statin therapy, muscle pain is associated with ‘statin intolerance’ whereby patients cannot take the drug at all or cannot escalate the dose sufficiently to reach treatment targets [10]. Whilst it has been clearly demonstrated that the vast majority of statin-associated muscle pain is attributable to the nocebo or drucebo effect (i.e. it results from the expectation of pain or misattribution rather than a pharmacological effect) [11, 12], long-term compliance with statin therapy remains a problem [13].

Furthermore, it is increasingly apparent that even optimal management of LDL-C does not eliminate the risk of CV events. This residual risk results in part from inflammation [14, 15] and is amenable to pharmacological interventions [16, 17], thereby revealing new approaches to the management of atherosclerosis, although not all the newly recognised drug targets are easily amenable to specific small-molecule therapeutic agents.

Finally, many small-molecule approaches have a ‘ceiling effect’, with biological redundancy or compensatory mechanisms limiting the maximum effect of the drug. For example, statin therapy results in upregulation of PCSK9 (the functions of which are described in greater detail below), limiting the extent of achievable LDL-C lowering [18••]. This can be a particular problem in specific groups of patients, such as those with familial hypercholesterolaemia (FH), who require a profound lowering of LDL-C to avoid the development of atherosclerotic disease [19–21].

Alternative approaches to targeting molecules important in the pathophysiology of atherosclerosis include monoclonal antibodies mAbs and RNA silencing techniques, which have the potential to overcome many of the limitations of ‘small-molecule’ approaches. Both approaches require injectable therapies and have the potential to result in longer lasting and more specific biological activities compared with orally active small molecules but differ in various respects. mAbs act predominantly by binding to extracellular proteins, whereas RNA-based therapies must enter cells to prevent the translation of mRNA. All examples of mAbs and RNA therapeutics used in humans to date require subcutaneous injections for administration and have the potential for injection site reactions. The effectiveness of monoclonal antibody therapies can be reduced by the development of autoantibodies, although such an effect has not yet been observed with RNA-based drugs. mAbs are comparatively complex and expensive to produce in comparison to RNA-based drugs [9].

## Inhibition of PCSK9 in the Prevention of Cardiovascular Disease

**Fig. 1:**
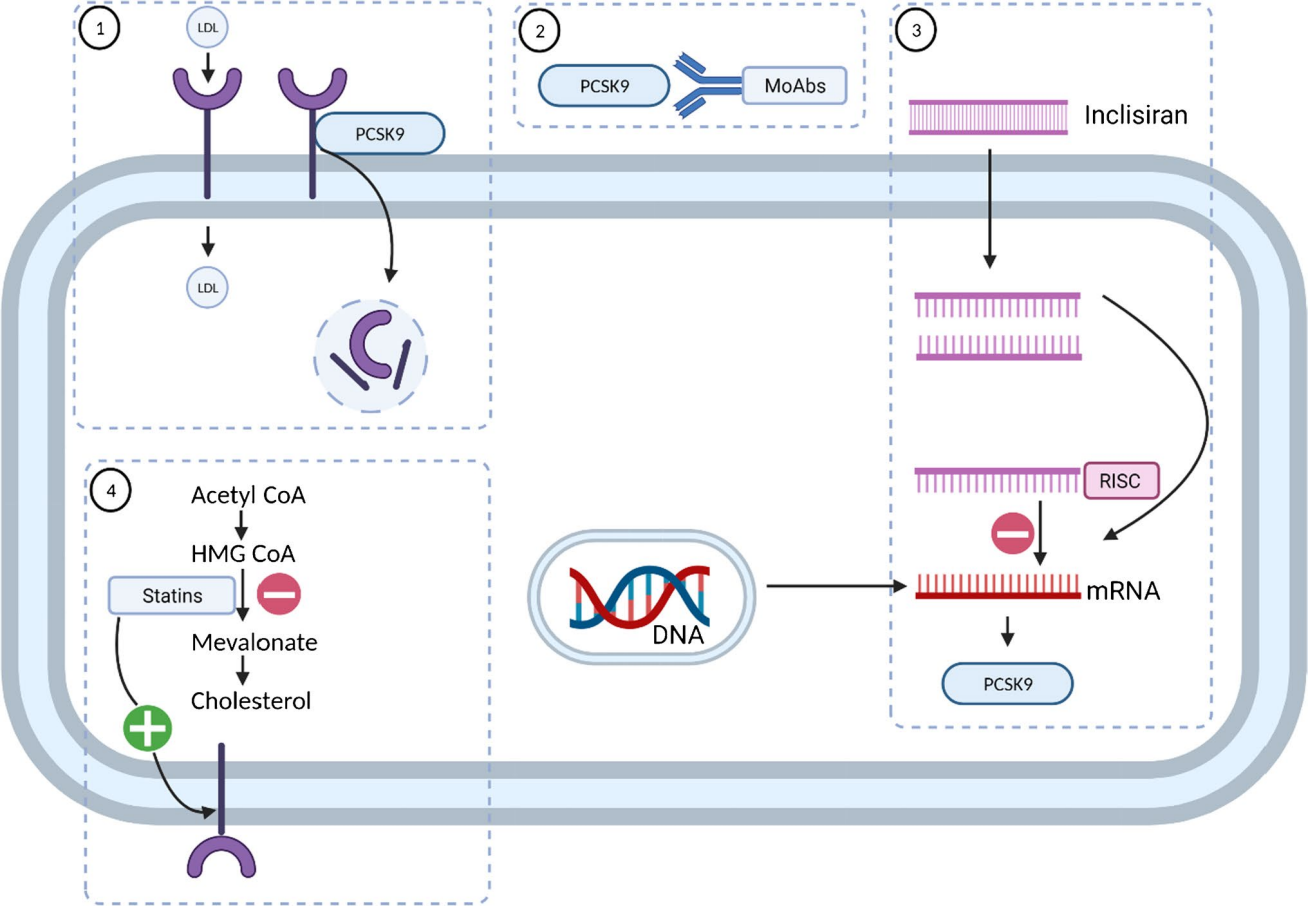
Mechanisms of action of lipid-lowering drugs in hepatocytes. (1) LDL is cleared from plasma through binding with LDL receptor (LDL-R) and internalisation; LDL-R binds to PCSK9 and is internalised and degraded. (2) Monoclonal antibody PCSK9 inhibitors (alirocumab and evolocumab) bind to PCSK9 and prevent its interaction with LDL-R, thereby increasing LDL-R on the cell surface. (3) Inclisiran produces long-lasting reduction in PCSK9 by silencing mRNA for PCSK9, thereby increasing LDL-R on the cell surface. (4) Statins reduce cholesterol production by inhibition of HMG-CoA reductase, the rate-limiting step in the mevalonate pathway; this leads to an upregulation of LDL-R on the cell surface. Abbreviations: CoA, coenzyme A; HMG-CoA, 3-hydroxy-3-methyl-glutaryl-coenzyme A; LDL, low-density lipoprotein; MoAbs, monoclonal antibodies; mRNA, messenger ribonucleic acid; PCSK9 proprotein convertase subtilisin kexin 9; RISC, RNA-induced silencing complex (figure was created with biorender.com)

PCSK9 has emerged in recent years as an important target in the management of atherosclerosis and has been targeted by monoclonal antibody drugs (evolocumab and alirocumab) [22••] and inclisiran [23], leading to substantial reductions in LDL-C. LDL is cleared from plasma by binding with hepatic LDL receptors (LDL-R), causing the lipoprotein particles to be taken up into hepatocytes. PCSK9 is a key regulator of LDL-R. The binding of LDL-R to PCSK9 results in internalisation and degradation of the receptor, reducing the capacity of the hepatocytes to take up LDL particles from the circulation. Monoclonal antibody PCSK9 inhibitors (alirocumab and evolocumab) bind to PCSK9 and prevent its interaction with LDL-R, thereby increasing the density of LDL-R on the cell surface. This approach has been demonstrated to reduce cardiovascular events in large randomised controlled trials [22••]. Inclisiran produces a long-lasting reduction in PCSK9 by silencing mRNA for PCSK9, thereby increasing LDL-R on the cell surface. Statins reduce cholesterol production by inhibition of HMG-CoA reductase, the rate-limiting step in the mevalonate pathway. The subsequent depletion of cholesterol in hepatocytes leads to an upregulation of LDL-R on the cell surface (Fig. 1).

## Inclisiran: Mechanism of Action

Inclisiran (previously designated as ALN-PCSsc, ALN-60212) is the first example of an approved synthetic small interfering RNA (siRNA) drug for treating FH [24]. The siRNA molecule inhibits the expression of PCSK9 by binding to the host mRNA strand encoding for the protein, causing it to cleave and degrade. This reduces the availability of the PCSK9 enzyme, which would otherwise block LDL receptors from initiating ingestion of LDL particles from the extracellular fluid. Inclisiran can therefore reduce blood concentration of LDL, with measurable clinical effects.

RNA interference (RNAi), as the biological basis for a subcategory of gene therapy, is a highly precise approach to suppressing the expression of specific genes [25]. This pathway is a cellular mechanism for defending against the effects of foreign genetic material being introduced into an organism, for example, through infection with a virus. RNAi can be exploited pharmaceutically to target and suppress genes that have been associated with diseases, with the added benefit of greater personalisation of therapeutics through sequence specificity [26].

RNAi can be achieved by two types of small double-stranded RNA molecule, namely small interfering RNA (siRNA) and microRNA (miRNA), both of which suppress gene expression according to their specific sequence albeit in slightly different ways: miRNA mediates the repression of mRNA translation, whereas siRNA mediates mRNA degradation. These differences provide a range of therapeutic possibilities, with the potential for one miRNA drug to target a number of different selected genes together, whereas an siRNA drug is able to target a single specific gene of interest. The focus of this review is on siRNA technology.

Once in the cytoplasm of a hepatocyte, the double-stranded siRNA drug inclisiran is uncoiled into two single, short RNA strands, one of which acts as the guide strand whilst the other becomes the passenger, which has no further role to play and is degraded [24, 27]. The inclisiran guide strand becomes bound to the RNA-induced silencing complex (RISC) and then pairs with the complementary mRNA strand for PCSK9. The RISC argonaute enzymes then cleave the PCSK9 mRNA sequence where it is bound to the corresponding inclisiran guide strand, effectively silencing the PCSK9 gene. With a reduced synthesis of the PCSK9 protein, LDL-C concentration in the blood plasma is reduced. Following mRNA cleavage, the inclisiran/RISC complex remains intact and retains its activity for further interference with gene expression of PCSK9, meaning that a single siRNA inclisiran molecule has some considerable long-term efficacy. Although only a small percentage of inclisiran administered becomes active in the hepatocytes, that which forms part of an inclisiran/RISC complex has a very long half-life allowing dosing in patients to be months apart [24].

As an alternative to double-stranded siRNA, RNA silencing can be achieved with single-stranded antisense oligonucleotides. These molecules ate typically 15–30 nucleotides in length and bind to mRNA, and prevent its translation, or cause its degradation [28].

Whilst siRNA therapeutics has been in development for around 20 years [29] for a number of different diseases, until recently, siRNA drugs have suffered drawbacks in terms of chemical stability and drug delivery [30, 31], which has prevented some promising candidates from becoming clinically useful. Simple, unmodified siRNA tends to be unstable, being susceptible to enzymatic degradation by RNases, and following injection, siRNA molecules are destroyed by endonucleases and exonucleases before they can reach the target cells [32]. Off-target effects are also possible since the RISC will allow a small number of sequence mismatches with the siRNA guide strand, meaning that unrelated genes with very similar sequences may also be silenced. Therefore, chemical modification and drug delivery methods have been developed in attempts to improve the overall acceptability of siRNA technology in the clinic.

Drug delivery technology has generally been improved, partly to protect the otherwise naked siRNA drug and partly to overcome obstacles in the way of reaching the intended target. In an early siRNA drug known as ALN-PCS, which targeted the PCSK9 gene by encapsulating the drug within lipid nanoparticles, it was possible to increase the drug half-life and increase endocytosis into target cells [33]. RNA drugs are large molecules, when compared with typical small-molecule compounds, and also carry a negative electrical charge—both properties decrease the likelihood of entering the cell across the lipid bilayer. Therefore, lipid nanoparticle encapsulation helps to overcome these drawbacks and aids intracellular delivery [34]. And because the liver is the primary filter for nanoparticles [35], it is possible to design novel siRNA drugs for dyslipidaemia that can target hepatocytes through this means [36].

Chemical conjugation is another method that has been employed to improve the stability and delivery of siRNAs. A covalently bound conjugate allows the siRNA drug to be targeted to specific tissues or cells by binding to a cell surface receptor, followed by entry through receptor-mediated endocytosis. Following on from ALN-PCS, an siRNA for PCSK9, known as ALN-PCSsc, was conjugated with N-acetylgalactosamine (GalNAc), which allowed the drug to target hepatocytes via the asialoglycoprotein receptor [37, 38]. The siRNA in inclisiran is also conjugated with GalNAc, building on this earlier approach [24].

## Inclisiran: Clinical Evidence and Guidelines

Inclisiran has been evaluated in a range of clinical trials (Table 1), including secondary prevention populations, high-risk primary prevention and individuals with homozygous FH (HoFH) or heterozygous FH (HeFH) [23]. Characteristics of ongoing and completed trials are summarised in Table 1, with their results (where available). Pooled analyses of currently available data are reassuring with respect to the safety and efficacy of inclisiran.

**Table 1:** Summary of clinical trials evaluating inclisiran

		Phase	Dates	Participants (n)	Population	Primary outcome	Results
ORION-1	NCT02597127	II	2016–2017	501	High CV risk and elevated LDL-C	% change in LDL-C	LDL-C ↓ between 29.5% and 46.4% [39]
ORION-2	NCT02963311	II	2016–2018	5	HoFH	% change in LDL-C	LDL-C ↓ PCSK9 ↓ [40]
ORION-3	NCT03060577	II	2017–2022		High CV risk and elevated LDL-C	% change in LDL-C	Not available
ORION-4 (HPS-4/TIMI 65)	NCT03705234	III	2018–2026	15,000	ASCVD	MACE	Not available
ORION-5	NCT03851705	III	2020–2021	56	HoFH	% change in LDL-C	Not available
ORION-7	NCT03159416	I	2017–2018	31	Comparing patients with renal impairment and normal renal function	Pharmacokinetic parameters	Inclisiran safe in renal impairment [41]
ORION-8	NCT03814187	III	2019–2023	2991	High CV risk and elevated LDL-C	Proportion of patients reaching LDL-C goal (< 70 mg/dl)	Not available
ORION-9	NCT03397121	III	2017–2019	482	HeFH	% change in LDL-C	LDL-C ↓ 53.8%[42]
ORION-10	NCT03399370	III	2017–2019	1561	ASCVD and elevated LDL-C	Absolute change in LDL-C	LDL-C ↓ 47.2%[43]
ORION-11	NCT03400800	III	2017–2019	1617	ASCVD or risk equivalents and elevated LDL-C	Absolute change in LDL-C	LDL-C ↓ 47.9%[43]
ORION-13	NCT04659863	III	2021–2023	15	Adolescent HoFH patients	% change in LDL-C	Not available
ORION-14	NCT04774003	I	2021	40	Chinese patients with elevated LDL-C	Pharmacokinetic parameters	Not available
ORION-16	NCT04652726	III	2021–2023	150	Adolescent HeFH patients	% change in LDL-C	Not available
V-INCEPTION	NCT04873934	III	2021–2023	384	Recent ACS and LDL-C > 70 mg/dl	% change in LDL-C	Not available
SPIRIT	NCT04807400	III	2021–2022	900	Patients on established LLT or have previously not tolerated LLT	% change in LDL-C	Not available
NA	NCT04666298	II	2021–2022	308	Japanese patients with high cardiovascular risk and elevated LDL-C	% change in LDL-C	Not available
NA	NCT04765657	III	2021–2022	320	Asian patients with ASCVD or risk equivalents and elevated LDL-C	% change in LDL-C	Not available
NA	NCT02314442	I	2015–2016	70	Healthy volunteers	Adverse events	No serious adverse events [24]
NA	NCT01437059	I	2011–2012	32	Healthy volunteers	Adverse events	No serious adverse events LDL-C ↓ 40% PCSK9 ↓ 70% [44]

Abbreviations: *ASCVD* atherosclerotic cardiovascular disease; *CV* cardiovascular; *HeFH* heterozygous familial hypercholesterolaemia, *HoFH* homozygous familial hypercholesterolaemia; *LLT* lipid-lowering therapy; *LDL-C* low-density lipoprotein cholesterol; *MACE* major adverse cardiovascular events

A patient-level pooled analysis including 3,660 patients from three ORION trials has recently been published. The analysis included data from patients with familial hypercholesterolaemia (ORION-9), ASCVD (ORION-10) and ASCVD risk equivalents (ORION-11); 92% of subjects were taking statin therapy and 14% were using ezetimibe. Patients were injected with inclisiran or placebo at baseline, after 3 months and then every 6 months. At 510 days, LDL-C was found to be reduced by 50.7% [95% confidence intervals − 48.9, 52.1%] in the inclisiran group, after correction for placebo. Adverse effects included bronchitis and mild injection site reactions.

A pre-specified safety analysis of the ORION-1 trial (501 patients) focused on haematological variables during a year of follow-up. No evidence was found that inclisiran affected counts of leucocytes, monocytes, platelets or neutrophils [45, 46]. Inflammatory markers (including IL6 and TNF-alpha) were not affected by inclisiran treatment. Provided these observations are replicated in larger trials, the drug is likely to have a very good safety profile.

Outcomes data available to date are extremely promising. A meta-analysis including data from ORION-10, ORION-11 and studies investigating MoAb-based PCSK9 inhibitors found that the relationship between LDL-C lowering and reduction in MACE followed the same trend for inclisiran and the MoAb agents [47]. A separate study pooling the results of ORION-9, ORION-10 and ORION-11 (a total of 3,660 patients) demonstrated a 24% reduction in MACE with inclisiran treatment, compared to placebo [48]. A particular advantage of inclisiran over other lipid-lowering therapies is the fact that it can be safely used in patients with mild (CrCl 60–89 mL/min), moderate (CrCl 30–59 mL/min) or severe (CrCl 30–59 mL/min) renal function impairment, without the need for dose adjustment, thereby addressing an unmet need, particularly in the secondary prevention of cardiovascular disease [41].

On the basis of these data, inclisiran is likely to play an increasing role in guideline-directed management of dyslipidaemias. Polish guidelines recommend that inclisiran may be considered in patients with ASCFD or FH who do not achieve lipid targets on statin and ezetimibe, in statin intolerance and in very high-risk primary prevention patients who do not adhere to, or consent to, other lipid-lowering therapies [49]. In the UK, the National Institute for Health and Care Excellence (NICE) is currently evaluating inclisiran for the treatment of primary hypercholesterolaemia or mixed dyslipidaemia [50]. Current recommendations and marketing approvals are based on the data showing robust long-term reduction of circulating LDL-C by inclisiran. More widespread use of inclisiran may be expected if it is demonstrated to reduce hard clinical outcomes to the extent that would be expected, given the success of MoAb-based PCSK9 inhibitors. The ongoing HPS-4/TIMI 65/ORION-4 double-blind randomised controlled clinical trial (NCT03705234) will provide important information in this respect. The study is currently recruiting 15,000 individuals with ASCVD at 180 clinical sites in the UK and the USA. Participants will be randomised in a 1:1 ratio to receive a placebo injection, or subcutaneous injection of inclisiran sodium 300 mg will be administered as at randomisation, 3 months and then every 6 months, and will be followed up for 5 years with a primary composite endpoint of the first occurrence of coronary heart disease death; myocardial infarction; fatal or non-fatal ischemic stroke; or urgent coronary revascularisation procedures (Table 1) [51].

Whilst inclisiran is the first drug in its class for dyslipidaemia, it is unlikely to be the only one since RNA inhibition offers, at least theoretically, the opportunity to target any gene of interest. The siRNA templates are already established, and therefore, it is a simple step to modify the sequence of nucleic acids to a specific target mRNA. Other proteins associated with plasma lipid homeostasis, particularly if they are functional in the liver, are therefore likely to be included in research and development programmes using this technology. An orally available antisense oligonucleotide for PCSK9 has recently demonstrated promise in preclinical studies [52], and RNA silencing is currently under investigation to target a range of proteins implicated in the pathophysiology of atherosclerosis (Table 2).

**Table 2:** RNA silencing therapeutics in development or use for dyslipidaemias

Target	Rationale	RNA silencing agent	Silencing method	Developmental stage
		AKCEA-APO(a)_RX_ IONIS-APO(a)_RX_	Antisense oligonucleotide (GalNAc complex)	Demonstrated Lp(a) reduction in Phase II [53]
		AMG890 Olpasiran	siRNA	Phase I (NCT03626662) Phase II (NCT04270760)
Apolipoprotein CIII	Lowers serum triglycerides	Volanesorsen	Antisense oligonucleotide	In clinical use
		AKCEA-APOCIII-L_RX_	Antisense oligonucleotide	Phase III (NCT04568434)
Apolipoprotein B	Apolipoprotein B is an essential component of atherogenic lipoproteins, including LDL	Mipomersen	Antisense oligonucleotide	In clinical use
ANGPTL3	ANGPTL3 inhibition reduces triglyceride and LDL and lowers cardiovascular risk	AKCEA-ANGPTL3-L_RX_	GalNAc-conjugated antisense oligonucleotide	Improved lipid profile in phase I trial [54] Awaiting results of phase II trial (NCT03371355)
PCSK9	PCSK9 inhibition increases LDL-R density on hepatocytes and improves LDL particle clearance from the blood	Inclisiran	siRNA	Phase III [23]
		AZD8233	Antisense oligonucleotide (orally available)	Preclinical studies [52]

## Conclusions

Pooled analysis of three recent ORION trials has demonstrated that twice-yearly administration of inclisiran reduces LDL-C by 50% in a range of patient groups, with only mild adverse effects. Inclisiran, therefore, is a safe, effective approach to the reduction of LDL-C. The results of the phase-3 ORION-4 outcomes study are eagerly awaited and will demonstrate the effectiveness of inclisiran against hard clinical outcomes. Further promising RNA silencing technologies directed at a variety of biological targets have the potential to improve the management of dyslipidaemias.
